# The psychology of the internet fraud victimization of older adults: A systematic review

**DOI:** 10.3389/fpsyg.2022.912242

**Published:** 2022-09-05

**Authors:** Yuxi Shang, Zhongxian Wu, Xiaoyu Du, Yanbin Jiang, Beibei Ma, Meihong Chi

**Affiliations:** ^1^School of Law, Shandong Normal University, Jinan, China; ^2^School of Psychology, Shandong Normal University, Jinan, China; ^3^Medicine, Dentistry and Health Sciences, University of Melbourne, Melbourne, VIC, Australia; ^4^School of Culture and Communication, Guangxi Science and Technology Normal University, Laibin, China

**Keywords:** older adults, internet fraud victims, cognitive function, personality traits, technology and experience

## Abstract

Criminals targeting and exploiting older adults in online environments are of great concern. This study systematically retrieved and analyzed articles on the psychological characteristics of older adult victims of online fraud. First, we found that there was no evidence that older adults were more prevalent than other individuals of other ages among online fraud victims, and current researchers have focused more on why older adults are easy targets for fraud (susceptibility to being cheated). Second, research on psychological factors of older adults' susceptibility to online fraud has mainly focused on cognitive function, trust traits, and other personality traits, such as social loneliness, the Big Five personality traits, and self-control. Among them, most researchers claim that the cyber-cheating of older adults may be due to a decline in their cognitive function. However, there has not been a consensus on how cognitive function and physical and mental conditions affect older people who are cheated. Third, techniques (i.e., methods and techniques used by fraudsters) and experience (i.e., familiarity with internet technology or fraud) may be related to the susceptibility of older adults to fraud, and these studies have also not yet generated a consensus supported by reliable data. Based on the above research uncertainties, we propose that fraud prevention and control strategies for older adults should be applied with caution.

## Introduction

Internet-based fraud targeting older adults is an emerging public health problem and a critical social problem in modern society (Ross et al., 2014; Lichtenberg et al., 2016; Yan et al., 2021). The reported financial losses of people over 55 years old, who are less well educated, more socially isolated and particularly vulnerable to scams, are nearly double those of the youngest people at each occurrence of online fraud in the UK (Fischer et al., 2013). Fraud activities can cause irreversible financial losses (Kircanski et al., 2018) and can also lead to negative emotions, such as depression and anxiety, and psychological pressure, such as anger, self-blame and shame, in older adult victims (Button et al., 2014). Why do millions of older adults continue to encounter online fraud despite rigorous, comprehensive programs designed to prevent and control fraudulent internet activities, widespread education on the use of technology and other methods to avoid being defrauded? Are older adults more likely to become victims of fraud?

Scholars first explain older adults' susceptibility to fraud from a demographic perspective. James et al. (2014) found that age is positively correlated with the level of susceptibility to fraud, especially as reported by news and media, and older adults are more likely to be victims. The susceptibility of older adults to fraud also varies with demographic features, such as income level (Lichtenberg et al., 2013; Gavett et al., 2017) and education level (Boyle et al., 2012). Based on laboratory research, there are at least six hypotheses about why older adults are disproportionately victimized by fraud (Ross et al., 2014). One of the most widely cited explanations is *the following:*

*Relative to younger adults, older adults exhibit less accurate episodic memories and increased vulnerability to misinformation (Grady and Craik*, *2000**; Jacoby and Rhodes*, *2006**). The AARP suggests that older adults are more susceptible to consumer fraud, in part, because of such age-related memory changes. For example, older persons might be more accepting of false claims about the past, such as “you forgot to pay me” (Kirchheimer*, *2011**)*.

Interpersonal fraud theory (Buller and Burgoon, 1996) explains the model of fraud formation for all people, including older adults. From this perspective, personal factors (e.g., goals, motivations, emotions, cognitive abilities) are necessary to predict and explain fraud (internet fraud). According to traditional fraud theory (Johnson et al., 2001; Zhang, 2016), the victimization process in online fraud comprises four stages (fraudulent online messages → assessment of information authenticity → trust generation → decision-making errors). Individuals' attention and processing patterns toward fraud clues are related to the likelihood of success in this four-stage fraud theory (Gao., 2021). This is consistent with the elaborate processing possibility model (ELM). In cognitive processing theory, individuals make decisions by diligently and systematically processing information or by relying on cognitive rules triggered by heuristic cues in the decision-making context (Chaiken and Eagly, 1989).

Current research on the susceptibility of older adults to online fraud has focused on individual psychological differences (Fischer et al., 2013; Button et al., 2014). Previous studies have found that the susceptibility of older adults to online fraud might be connected to their mental health status (Lichtenberg et al., 2013; James et al., 2014), cognitive ability (Judges, 2017), extroversion level (Reisig and Holtfreter, 2013), trust level (Han et al., 2016), self-control (Holtfreter et al., 2008), or security and perceived control (Yang et al., 2019b). For example, the results of a survey of 255 older people by (Yang et al., 2019b) showed that aging fear influences gullibility, and the sense of security and control may be one of the internal mechanisms by which aging fear affects the gullibility of older adults.

The susceptibility to persuasion scale (e.g., Modic, 2012; James et al., 2014) was used to measure the factors affecting older adults' susceptibility to persuasion. James et al. (2012) measured 639 community-dwelling older adults without dementia through the susceptibility to persuasion scale and found that older age, lower levels of cognitive function, decreased psychological wellbeing, and lower literacy in particular may be markers of susceptibility to financial victimization in old age. The results of the susceptibility to persuasion scale also revealed that credulity rather than general trust may increase vulnerability to cyber fraud in older adults (Shao et al., 2019).

Nonetheless, some previous research has shown that although aging may lead to cognitive deterioration, age and job performance indicators are not always positively associated, and increasing age does not always predict a higher possibility of being deceived (Salthouse, 2012). By reviewing evidence on the prevalence of consumer fraud, Ross et al. (2014) suggested that psychologists may underestimate the influence of possible protective factors associated with old age in everyday life, including experience gain and goals, lifestyle, income, buying and risk behaviors. Therefore, the conclusion that fraud is more common among older adults is premature and lacks sufficient evidence to support it.

However, it cannot be denied that the overwhelming majority of studies support that older adults have more susceptibility factors among victims of online fraud, especially psychological factors (Jensen, 2011; Kirchheimer, 2011; Sarno et al., 2020). Psychological factors are specifically individual traits (or individual tendencies), the relatively stable, consistent and persistent internal characteristics of individuals in their behaviors, attitudes, feelings and habits, which can describe or determine the behaviors of individuals in various scenarios and situations (APA style, 2022). Psychologists claim that psychological traits have an impact on decision making in online fraud scenarios (Gao., 2021).

The present study retrieved and analyzed literature related to the psychological features of older adult victims of internet fraud, with the goal of collating and discussing previous research conclusions and further promoting exploration in this field. Of course, we also explained the unsupported views in the results section.

## Methods

### Systematic review

Strictly speaking, our manuscript is a systematic review rather than a meta-analysis, mainly because psychological factors, as mentioned above, involve trust, cognitive ability, personality and many other factors. However, few studies have focused on a specific factor. In addition, most current studies on the psychological factors of online fraud against older adults rarely adopt the method of controlled experiments, and there was no discussion of specific prevention and control strategies. The above reasons make it difficult for our analysis to meet the prerequisite conditions of a meta-analysis (Cheung and Vijayakumar, 2016).

Although it is difficult to perform a meta-analysis, our systematic review was conducted using the guidelines and checklist outlined by the Preferred Reporting Items for Systematic reviews and Meta-Analyses (PRISMA) group (Moher et al., 2009). Accordingly, our main research question describing the Population, Intervention, Comparison, Outcomes, and Study design (PICOS) involved samples composed of older adult victims who have suffered from online fraud (P) and various interventions designed for online older adult fraud victims (I) (if they existed) compared to control conditions (C) (if they existed) and outcomes related to the psychological factors of older adult victims of online fraud (O) in randomized controlled trials (S) (if they existed).

### Search strategy

This study included a systematic search of the high-quality literature using the PRISMA guidelines based on relevant full-text articles selected from multiple database searches of all published documents from the time of each database's establishment until May 2021 (updated search process on 16 May 2022). The following English-language databases were searched: ProQuest, Elsevier, EBSCO/ASC&BSC, Web of Science, and PsycArticles. The English search strategy was as follows: (old adults OR the older adult OR older people OR older person OR aging) AND (fraud OR cheat OR swindle OR scams OR deception OR susceptibility to scams OR phishing vulnerability OR susceptibility to persuasion) AND (psychology OR personality OR cognition OR information processing OR trust OR responsibility OR self-control OR physical condition OR physical weakness OR disability OR memory) AND (phishing emails OR phishing OR phished OR online OR internet OR cyber OR telemarketing).

Because there are various types of online fraud, such as grandparent scams, romance scams, phishing, and social networking fraud, we have diversified processing in setting the keywords of online fraud. The terms “online fraud” and “psychological factors” are set up the same way.

**Figure 1:**
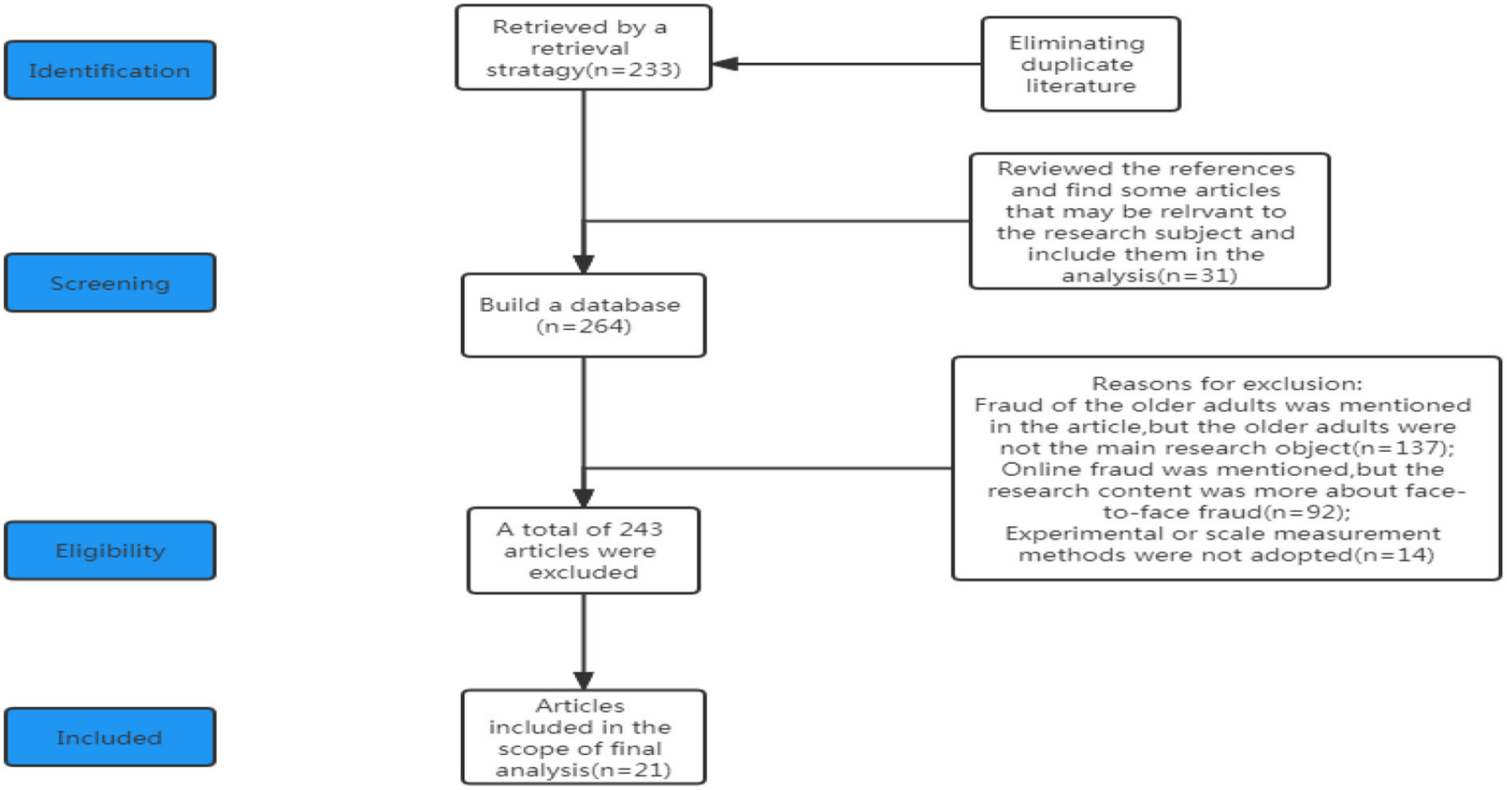
Identification flowchart of the search for psychological literature on older adult victims of online fraud (model drawing comes from the author's construction).

In addition, to improve the retrieval rate, we manually searched the literature for references, and the quality evaluation was carried out independently by two authors. We subsequently executed backward (e.g., reference lists of eligible studies) and forward searches (e.g., articles that cited the eligible studies using Google Scholar) for completeness. Figure 1 is the flowchart outlining the search and exclusion process for selecting preferred reporting items according to the meta-analysis (PRISMA) guidelines and systematic evaluation.

### Inclusion criteria and exclusion criteria

#### Article type

We considered primary studies for inclusion if they (i) conducted the experimental studies by the scale measurement and (ii) randomized participants to the experimental conditions. We excluded studies if they (i) had a non-experimental design (e.g., interpretive phenomenological analysis, anecdotal comments on cases and scams, cross-sectional); (ii) were written in any language other than English; (iii) lacked the availability of the full text via our university library subscriptions or directly from the corresponding author; (iv) were quasi-experimental, such as a non-random assignment; or (v) were published in abstract form (not full text).

#### Research population

Older adults were the focus of the preliminary analysis. The age standard for older adults varies by country, so we do not define the range of older people. Studies were included in our scope of review as long as the experimental population was represented in terms of older adults. If older people were not the subjects of the experiment, for example, but only mentioned in the article, it was not included in our analysis scope. There were no restrictions on the type of older adult demographics.

#### Research content

It should be noted that online fraud involves the use of network communication technology to obtain money through deception (Whitty, 2015, 2019) or the use of the internet to offer fraudulent invitations or conduct fraudulent transactions involving potential victims (Tade and Aliyu, 2011). In other words, unlike traditional scams, online scams do not occur in person. Studies that did not focus on online fraud were excluded from our review.

The psychological factors of online fraud are individual traits, including trust, cognitive ability, personality and many other factors (APA style, 2022). If the research did not focus on psychological factors or a particular trait, it was not included in our review.

#### Results of the research

Published papers needed to provide enough information to calculate the effect of psychological factors on fraud toward older adults. Results that were published only as conference abstracts were excluded from the review because they were incomplete.

### Article screening

The articles in the database were independently reviewed by two reviewers. Under the guidance of the search strategy, a total of 233 relevant articles were obtained after eliminating the repeated articles. First, we reviewed the references of these 233 articles, found that 31 additional articles might be related to the research subject, and established a database containing 264 articles. Second, according to the inclusion and exclusion criteria, we reviewed the entire text of the 264 articles, and 21 articles were included in our analysis scope. A total of 136 articles were excluded because older adults were not the main research objects, although older adults were mentioned in the articles; 92 articles mentioned online fraud, but the research content was more about face-to-face fraud, so those articles were also eliminated; and 15 articles that did not adopt the experimental or scale measurement methods (non-empirical methods, e.g., literature reviews, or phenomenon reports) were also excluded. See Figure 1 for the selection process.

### Risk of bias

**Figure 2:**
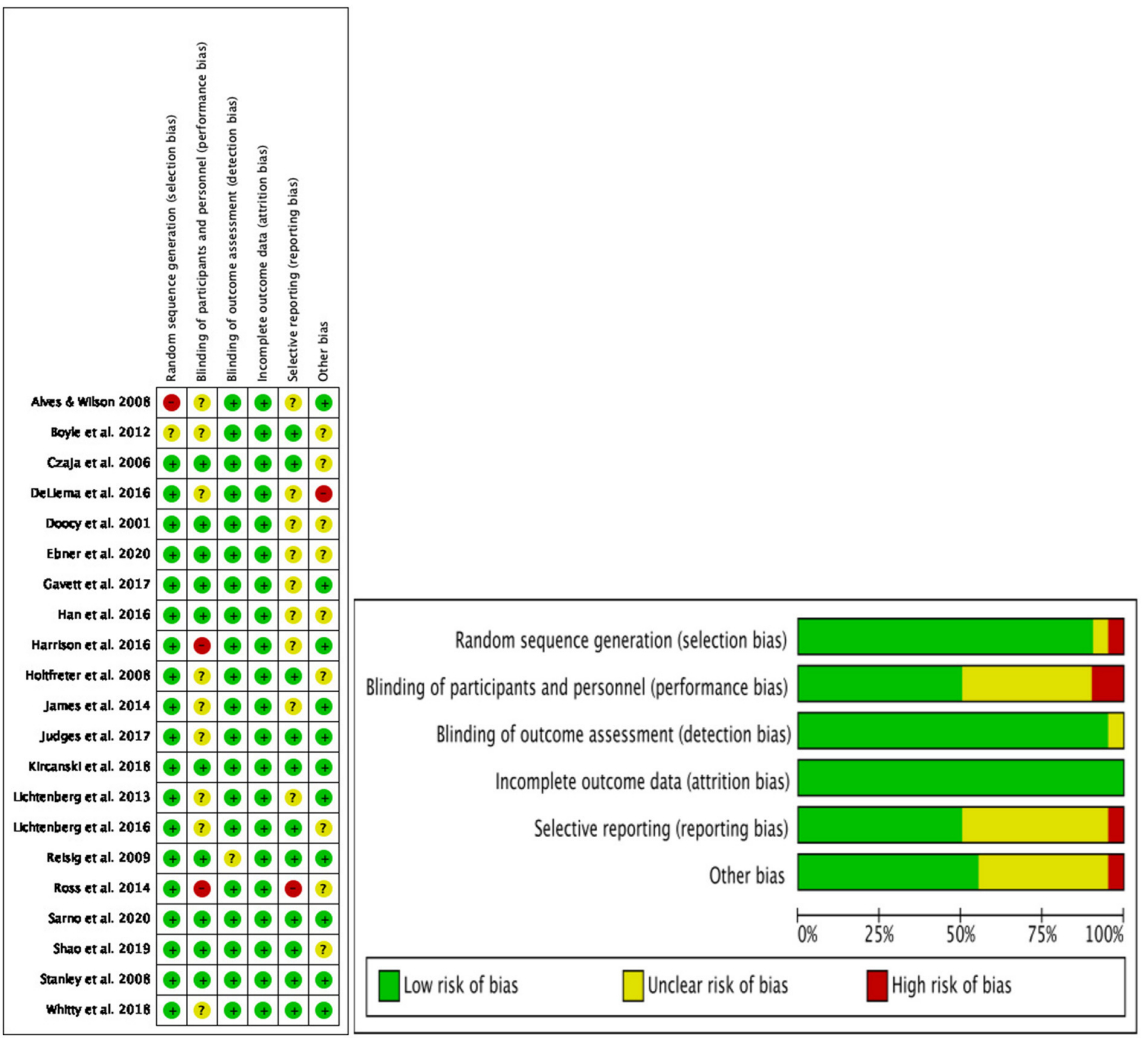
Risk of bias for included studies in the preliminary analysis for psychological literature on older adult victims of online fraud.

Although our paper is a systematic review, not a strict meta-analysis, we used the Cochrane risk of bias tool to evaluate the risk of bias for the included studies (Armijo-Olivo et al., 2012). The risk of bias of the individual studies was examined across six domains: selection bias, reporting bias, performance bias, detection bias, attrition bias and other bias (publication bias). We used a randomization strategy to address selection bias. We checked for whether the studies reported their outcomes of interest and related outcomes for the study in response to a selective outcome (reporting bias). Whether the participants were aware of the intervention was assessed to determine whether there was performance bias. We reviewed whether the measurement or ascertainment of the outcome differed between intervention groups due to detection bias. For attrition bias, we primarily examined whether the authors reported data loss. For publication bias, we mainly looked for high-quality papers published in influential national journals. To ensure judgement consistency across all studies, two members independently assessed study quality and then resolved conflicts together. A summary of all primary studies is depicted in Figure 2.

## Results

The Supplementary Table shows the characteristics of studies (N = 21) on older adult victims of internet fraud. We included a diverse set of studies, related methods, samples, and key findings. The researchers conducted the studies in the United States (N = 17) (Doocy et al., 2001; Alves and Wilson, 2008; Holtfreter et al., 2008; Stanley and Blanchard-Fields, 2008; Reisig et al., 2009; Boyle et al., 2012; Atkins and Huang, 2013; Lichtenberg et al., 2013, 2016; James et al., 2014; Han et al., 2016; Harrison et al., 2016; Gavett et al., 2017; DeLiema, 2018; Kircanski et al., 2018; Ebner et al., 2020; Sarno et al., 2020), Canada (N = 2) (Ross et al., 2014; Judges, 2017), the UK (N = 1) (Whitty, 2018), and China (N = 1) (Shao et al., 2019). Experimental methods were used in 12 of the 21 studies, and questionnaire surveys were used in the other 9 research projects (Supplementary Table).

Older adults are the key targets for efforts at preventing online fraud victimization (Kirchheimer, 2011; Gavett et al., 2017; Li, 2020), which prompts the shift of academic research from its traditional focus on fraudsters to its more recent focus on the individual related factors of older adults. The review of the included literature found that researchers tended to claim that the factors, including psychological variables, the techniques of fraudsters, and level of experience (e.g., network familiarity and fraud knowledge), affected the vulnerability of older adults.

### Psychological factors influencing the online deception of older adults

#### Cognitive function

Research on the cognitive factors of older adult victims of internet fraud is mainly based on the cognitive deterioration of older adults (Vishwanath et al., 2011; Han et al., 2015, 2016). Physiological aging is accompanied by cognitive aging. Some studies have found that in the absence of any obvious neurological or psychiatric diseases, older adults show a systemic decline in memory, processing speed, problem-solving ability, mathematical skills, linguistic ability, and executive function (Felson and Cohen, 1980; Murphy et al., 2006; Kvavilashvili et al., 2009; Ebner et al., 2020). The ability to accurately identify online fraud information is grounded in a high level of cognitive function. The fraud susceptibility of older adults is negatively related to their cognitive function and the level of each specific cognitive domain (Pinsker and McFarland, 2010; Boyle et al., 2012; Li et al., 2013; James et al., 2014). For example, Boyle et al. (2012) found that “decision-making ability is negatively correlated (*r* = −0.26, *p* < 0.001) with age, positively correlated (*r* = 0.58, *p* < 0.001) with overall cognition, and the susceptibility to fraud is negatively correlated (*r* = −0.30, *p* < 0.001) with overall cognition”. Other scholars previously found that the decline in the cognitive ability of older adults leads them to avoid negative information in their initial attention, leading them to be cheated because they cannot recognize the fraud of the information (Charles et al., 2003; Mather and Carstensen, 2003). The faster the deterioration of cognitive function occurs, the worse the decision-making ability becomes, and the more vulnerable the person is to being deceived. Many studies that have concentrated on exploring older adult victims of internet-based fraud from the perspectives of specific cognitive fields have found that lesions in the ventromedial prefrontal lobe, low levels of fluid intelligence (intelligence is divided into fluid intelligence and crystallized intelligence; fluid intelligence is a kind of physiology-based cognitive ability; the development of fluid intelligence is closely related to age, and it decreases with age after 30), poor visual and audiovisual performance, and neurocognitive and social cognitive deficits can impact victims' susceptibility to fraud (Czaja et al., 2006; Reisig et al., 2009; Asp et al., 2012; James et al., 2014).

Additionally, ELM is one of the important theories to explain the vulnerability of older adults to online fraud because it can simultaneously verify that the victim's attention and processing methods to fraud signals can determine whether he or she will be cheated Harrison et al. (2016). Han et al. (2016) found that the low level of specific cognitive abilities of older adults with mild cognitive impairment, such as perception speed and cognitive memory, are more inclined to reason using a heuristic decision-making model that saves mental resources, which may explain why they are more easily deceived online (*B* = 0.125, *p* < 0.05).

However, a few studies have also shown that the degradation of individuals' cognitive function might not be a reasonable explanation for why older adults become victims of online fraud. Among them, the strongest objection came from Ross, who argued that it is a false proposition that older adults are more vulnerable to fraud when the evidence of cognition is prospective memory (remembering to perform intended tasks); younger adults perform better than older adults on prospective memory tasks in the laboratory, but older adults exhibit superior prospective memory in everyday life (Phillips et al., 2008; Ross et al., 2014). There is also a view that although there is cognitive decline with age, the effect of this condition on the daily lives of older adults is not significant (Salthouse, 2012). Older people may also choose to respond to cognitive decline with more cautious judgements to avoid being tricked (Sarno et al., 2020). To prevent fraud activities, Ross et al. (2014) proposed that experience is more helpful than cognition.

Although previous studies investigating the relationship between cognitive deterioration and fraud susceptibility among older adults have not yet reached a consensus, more than 60% of the studies related to fraud susceptibility have concluded that cognitive deterioration is the internal mechanism of online fraud susceptibility in older adults.

#### Associated variables of cognitive function

Practically speaking, researchers have explored not only the predictive effect of cognitive deterioration on online fraud susceptibility among older adults but also the factors that may lead to a decline in cognitive ability. The discussions have focused mainly on how mental health and physical conditions affect the susceptibility of older adults to online fraud through intermediate variables such as cognitive ability (Alves and Wilson, 2008; Lichtenberg et al., 2013; James et al., 2014).

#### Mental health

The most powerful psychological factor associated with online fraud susceptibility among older adults is mental health status (Lichtenberg et al., 2016), and the degree of depression is positively correlated with fraud susceptibility (*p* = 0.03) (James et al., 2014). If older adults have a stable mood, a positive attitude toward their lives, and a healthy will, they are less likely to be used by fraudsters (Lichtenberg et al., 2013; James et al., 2014). *Late-life depression* (LLD) is a common mental disorder associated with severe disability and cognitive impairment. The depression variable may directly or indirectly affect the susceptibility of older adults to fraud through the mediation of cognitive variables. Geriatric depression is a common mental disorder associated with severe disability and cognitive impairment, and up to 60% of the population with LLD shows mild cognitive impairment, with executive dysfunction and vulnerability in memory, information processing speed, attention, language, and visuospatial abilities (Butters et al., 2004). Crocco et al. (2010) discussed how LLD affects episodic memory and short-term memory in older adults from the aspect of neural mechanisms. However, lower levels of short-term episodic memory were associated with higher susceptibility to online fraud in older adults (*B* = −1.38, *p* = 0.01) (Ebner et al., 2020).

It is worth noting that among older adults, the need for status decreases with advancing age while emotional needs increase, and if there is no continuous behavior confirmation, depression is more likely to occur (Lichtenberg et al., 2013). Moreover, as fewer social needs are met, more severe symptoms of depression develop. However, the researchers found that fraud prevalence was three times higher (14%) among those with the highest depression and the lowest social-needs fulfillment than among the rest of the sample (χ^2^ = 20.49, *p* < 0.001) (Lichtenberg et al., 2013). In other words, social engagement may not only affect the susceptibility to online fraud among older adults through multiple mediating effects such as depression and cognitive decline but also directly influence their susceptibility. However, this conclusion lacks direct evidence.

### Physical conditions

Some studies have claimed that the reduced living space caused by physical disability and handicaps among older adults could increase their risk of Alzheimer's disease, mild cognitive impairment, cognitive deterioration and depression (James et al., 2011; Lichtenberg et al., 2013). Pinsker and McFarland (2010) found that older adults with physical disabilities relied more on others for financial decision-making and management and thus may be more vulnerable to inappropriate influence and abuse.

The routine activity theory and the social vulnerability model of older adults proposed by Greenspan et al. (2001) also explain the high susceptibility of older adult victims. Greenspan et al. claimed that the fragility of older adults (including physically and mentally fragile older people) is responsible for their increased susceptibility to online fraud. Fragile older adults are more likely to be targeted by fraudsters (Friedman, 1992).

However, some studies also state that a worsening of the physical condition is not significantly related to fraud susceptibility among the older population. For example, James et al. (2014) failed to prove a correlation between health, disability and social integration variables and susceptibility to fraud, and they claim that healthy and active older adults seem to be as vulnerable to fraud as unhealthy and disabled old people (*p* = 0.34). DeLiema (2018) found that there were few significant differences in physical health and cognitive functioning at the time victims' assets were taken, although their social contexts were different (*p*_physicalhealth_ =0.369; *p*_memory_ =*0.1*43).

#### Trust traits and influencing variables

In research on the susceptibility of older adults to online fraud, trust is the most deeply discussed personality trait. Are older adults more easily deceived than younger people because they are more likely to trust others? Although this is just a guess, it is not unreasonable. Compared with other factors, experiments have proven that older adults show weaker anterior insula activation for untrustworthy faces, and they are more likely than young people to trust and be open to untrustworthy people. Therefore, at least part of the reason for older adults being more likely to become victims of online fraud is that they are less sensitive to untrustworthy clues because of their generally high level of trust in others (Castle et al., 2012). On the other hand, the opposite of trust is the questioning and suspicious personality type. As suspicion increases uncertainty, it prompts individuals to want a larger amount of and more accurate information before making judgements, thus enabling their analytical reasoning and decision-making systems to process all available information. However, older adults might be less suspicious due to their high levels of trust and affinity (affinity is the closeness of a person or an organization in the views of the masses, showing warmth and friendliness, Nakayama and Morimoto, 2007) for people, so they are more likely to be deceived (Harrison et al., 2016).

In addition, Shao et al. (2019) made a distinction between general trust and credulity, and credulity, not general trust, increased older adults' vulnerability to fraud. They pointed out that older adults tend to have a higher level of trust in their friends, neighbors and relatives, which increased their social defense mechanism. In contrast, credulity is a propensity to believe things that are unproven or unlikely to be true (Pinsker et al., 2010). Some theorists tend to use credulity to explain an individual's chance of suffering financial exploitation or fraud (Greenspan et al., 2001) because some older adults seem to have more difficulty recognizing potentially fraudulent practices or fraudulent situations (Pinsker and McFarland, 2010). Moreover, the study found that general trust is not significantly associated with vulnerability to fraud (*r* = 0.04, *p*> 0.05). However, credulity is positively associated with vulnerability to fraud (*r* = 0.49, *p* < 0.01) (Shao et al., 2019).

Despite the increase in age, the level of trust in older adults is also influenced by other related personality features. Many scholars have discussed how personality factors influence online fraud susceptibility through the trust variable. A study showed that people with high levels of affinity and agreeableness traits (agreeableness involves either possessing a pleasant disposition or conforming to others' wishes, Graziano and Eisenberg, 1997) were more likely to trust and were more cooperative with others, and they may also be more likely to respond to other people's information (Koole et al., 2001).

However, some scholars have put forward different opinions on the influence of trust factors on fraud susceptibility. Carter and Weber (2010) claimed that high-trust people are significantly better than low-trust people at detecting lies and are more likely to distinguish between trustworthy and untrustworthy people. Therefore, compared with the younger population, older adults are less likely to be deceived (Carter and Weber, 2010). Judges (2017) also suggested that there is no significant correlation (*p* = 0.37) between trust and fraud victimization, nor is there a significant correlation (*p* > 0.05) between affinity and susceptibility to fraud victimization (Pinsker and McFarland, 2010; James et al., 2014).

#### Other personality trait variables

Apart from the trust personality variable, researchers also discovered the relationships between other personality characteristics of the older population and their susceptibility to fraud. These personality traits include the “Big Five”, self-control, and social loneliness (Alseadoon et al., 2012; Iuga et al., 2016; Judges, 2017).

The Big Five are often used to explain the causes of internet fraud, but conscientiousness has been discussed in the literature on internet fraud against older adults. Older adults with high levels of responsibility might be more likely to get into the habit of checking email, hence improving the possibility of falling into the trap of phishing emails (Vishwanath et al., 2011). In fact, studies on ordinary victims of internet fraud have yet to find a correlation between conscientiousness and susceptibility (*p* > 0.05) (Judges, 2017).

The low self-control theory of Gottfredson and Hirschi (1990) states that people with low self-control are short-sighted and are less likely to take measures to protect themselves from potential harm while pursuing targeted activities. Therefore, low self-control could increase the possibility of older adults being defrauded. In addition, some researchers have found that an indirect effect on susceptibility to fraud on the fear of aging (worries and fears about social and interpersonal loss related to aging, Grenier et al., 2011) affects only older adults with low levels of self-control (β = 0.28, *p* < 0.001) (Holtfreter et al., 2008; Reisig and Holtfreter, 2013; Yang et al., 2019a).

Social isolation is a state of unmet social needs, and loneliness is a subjective feeling (Victor et al., 2002). Social isolation and loneliness are also related to the vulnerability of older adults to fraud. Research shows that the scores of older adults aged 60–70 in the Revised UCLA Loneliness Scale (RULS) and Emotional Social Loneliness Inventory (ESLI) are significantly higher than those of other groups. Similarly, this group accounts for the largest proportion of victims of telemarketing fraud (Alves and Wilson, 2008). Many researchers claim that socially isolated consumers are more likely to rely on marketers to meet their social needs for interaction and communication (Kang and Ridgway, 1996). Loneliness makes older adults more eager to establish social relationships with others, thereby increasing their desire to communicate with strangers and finally causing them to fall into the traps of liars (Alves and Wilson, 2008). Alves and Wilson (2008) found that a decline in health and loss of intimacy could increase loneliness in older adults, and increased loneliness could make older adults more susceptible to fraud (*r* = 0.92, *p* = 0.00).

### Empirical knowledge and technical factors influencing the online deception of older adults

Although psychological variables are the focus of research on online fraud susceptibility among older adults, previous studies have frequently discussed experience and techniques, which were mainly manifested in unfamiliarity with fraud technologies and lack of internet experience.

#### Technical factors

Fischer et al. (2013) summarized four characteristics of online fraud information that were frequently used by online fraudsters: high returns as bait; claims of official information; social influence, including liking and reciprocation, designed to gain compliance; and the scarcity and urgency of opportunities. Online fraudsters are systematically trained, that is, how to use communication skills to make potential victims confirm that the information is “true”, before they commit fraud, resulting in inappropriate decisions and property damage (Friestad and Wright, 1994; Burnes et al., 2017).

Previous studies have found that two main technical factors prompt older adults to respond to fraud. The first is trust and reliance on authority. The use of official logos and names increase trust among older adults, thereby making them more likely to comply with scams (Fischer et al., 2013; DeLiema et al., 2016). Older adult victims like formality and authority, and this simple and intuitive thought may make them relax and lose their vigilance (Huang, 2020). The second is the instinct trigger. Scammers trigger the instincts of older adults mainly by increasing their victims' motivation to respond by offering generous prizes or magical medicines (Doocy et al., 2001; Fischer et al., 2008; Ariely et al., 2009), emphasizing the scarcity and urgency of opportunities (Lynn, 1989; Fischer et al., 2013), playing to the desire for social identity and consistency (Festinger, 1957, 1964; Frey, 1986), and using stress and coercion (Fischer et al., 2013; Button et al., 2014). For example, the stimulus of urgent clues in emails or other scam channels pressures victims to accelerate information-processing decisions, thereby shortening the window to consult other resources that can help them detect deceptions, and the perception of scarcity increases the subjective value of scam bait and makes it easier for people to make incorrect decisions and be deceived (β = 0.23, *p* < 0.05) (Vishwanath et al., 2011).

Some researchers have found that deceivers may put the target in an “emotional ether” (high-arousal positive emotion, e.g., exhilaration, or high-arousal negative emotion, e.g., anger, fear) in the course of persuading older adults to be deceived (Loewenstein, 1996; Cukier et al., 2007; DeLiema et al., 2016). For older people, as they grow older, they may prioritize goals related to optimizing emotions and social experiences (Carstensen et al., 2006). Attention to the internal cause may make older adults execute emotion-related information processing (heuristic information-processing mode) and avoid analytical processing (Loewenstein, 2000; Ariely et al., 2009). For example, high emotional arousal is targeted to focus attention on reward cues associated with deception and reduce cognitive efforts to deal with phishing emails and reduce attention to indicators of deception (Langenderfer and Shimp, 2001; Wang et al., 2012).

In later experiments by Kircanski et al. (2018), it was found that in situations involving high positive emotions and high negative emotions, the participants increased their willingness to buy the falsely advertised product compared with situations involving high excited negative emotions. However, emotional arousal was not found to increase older adults' vulnerability to phishing attacks compared with younger people who were also emotionally aroused (*p*_highpositiveemotion_ = 0.530, *p*_highnegativeemotion_ = 0.645). The time pressure of decision-making was an important factor in inducing emotion in the process of older victims. Under time pressure, older people showed no significant difference in the accuracy of fraud identification of emails compared with younger people, although both were lower (*p* = 0.156) (Sarno et al., 2020).

#### Empirical knowledge factors

The successful adoption of technology is becoming increasingly important to functional independence. One finding indicated that older adults were less likely than younger adults to use technology in general. They had less familiarity and comfort with and lower self-efficacy in the use of computers and smartphones than younger people, which may reflect a lack of understanding of the internet (Czaja et al., 2006). According to the heuristic system model (HSM) proposed by Chen and Chaiken (1999) and ELM, older adults tend to rely on simpler search strategies and consider less information before making decisions due to a lack of network knowledge (Reisig et al., 2009; Mata and Nunes, 2010; Queen and Hess, 2010). Lack of experience may produce challenges for older adults when classifying email, and this might be a reason for the older population becoming a target of online fraud (Grimes et al., 2007). Diao and Zeng (2020) also confirmed the negative correlation between online self-efficacy and susceptibility to online fraud (*t* = 6. 23, *p* = 0.00).

However, educational background could be regarded as a proxy for familiarity and expertise with computers to a certain extent, and older people with more years of education tend to be more suspicious of phishing attempts (Gavett et al., 2017). As cognitive flexibility begins to decline in adulthood (Salthouse, 2004), older adults could have difficulty learning new things, such as the use of the internet. However, the growth of crystal intelligence caused by the experience of online fraud could compensate for this shortcoming (Czaja et al., 2006). Experience-based knowledge could help older adults make appropriate decisions without actively processing all information (β=0.47, *p* < 0.001) (Li et al., 2013). As mentioned in the introduction, studies have also shown that the increase in financial decision-making experience with age plays a greater role in preventing fraud than cognitive ability (James et al., 2012; Ross et al., 2014). In fact, Sarno et al. (2020) found that regardless of network experience, no age differences were observed in overall classification accuracy; rather, all participants exhibited poor performance (*p* = 0.156).

In addition, previous experiences of victimization might decrease individuals' fraud susceptibility. Older adults who have previously been victims of online fraud might have some understanding of it (related to prior knowledge). After being deceived, they may unconsciously improve their perception of financial risks. If this is the case, they will not easily fall into the trap of online fraud (Gavett et al., 2017).

## Discussion

### Internet fraud toward older adults: A matter of prevalence or susceptibility?

According to previous studies, we first need to discuss the question of whether older adults are more frequent victims of online fraud than other groups or more susceptible to fraud. In other words, is it a matter of prevalence or susceptibility?

Many of the arguments that older people are more likely to be targets of online fraud come from news reports. For example, Kirchheimer (2011) reported that adults older than 65 years of age constituted one-eighth of the U.S. population but as much as one-third of all victims of consumer fraud, and this was published in the AARP (formerly the American Association of Retired Persons) Bulletin. A recent meta-analysis also revealed that elder financial fraud and scams affect approximately 1 of every 18 cognitively intact, community-dwelling older adults each year and concluded “the prevalence of financial fraud and scams among older adults in the United States” (Burnes et al., 2017). Although the articles included in our analysis did not investigate the prevalence of online fraud among older adults, they mostly cited previous news reports (e.g., Shao et al., 2019).

Some scholars strongly challenged the conclusion that older people were more vulnerable to fraud. First, the claim that older adults were disproportionately vulnerable to online fraud was not supported by survey data (especially persistent data); even though there were previous news reports that supported the claim, those were self-reported and not scientific (Ross et al., 2014). In fact, Sarno et al. (2020) confirmed that there were no age-related differences in phishing vulnerability through experimental studies under various task conditions.

Therefore, it is premature to conclude that older adults are more vulnerable to online fraud. Strictly speaking, what scholars discuss is the susceptibility of older individuals to online fraud rather than the prevalence.

### Related factors affecting older adults' susceptibility to online fraud

#### Psychological variables

With increasing age, the fear of aging is generally present among older adults (Yang et al., 2019b). Relatedly, the overall social needs of older adults neither decrease nor even increase with age. Additionally, when their social needs are not met, older adults' loneliness and depression increase (DeLiema et al., 2016; Pan, 2016). Studies have found that physical aging reduces cognitive function among older adults (Kvavilashvili et al., 2009; Ross et al., 2014), while physical decline, increased loneliness, and fear of aging also weaken cognitive ability among older adults (James et al., 2011; Lichtenberg et al., 2013). Cognitive decline may make it difficult for older adults to recognize fraud techniques, making them more likely to fall into the traps of online fraudsters (Alves and Wilson, 2008; Stanley and Blanchard-Fields, 2008; James et al., 2014; Yang et al., 2019b). However, these studies do not clearly explain how scammers use these factors and how they affect the decision-making processes of older adults. According to the dual-system processing model, human decision-making is produced by processing information through two paths: heuristic processing and systematic processing (Chaiken and Eagly, 1989; Priester et al., 1999). The emergence of mental health problems and the decline in physiological functions among older adults are often accompanied by a decline in cognitive function (Friedman, 1992; Butters et al., 2004), which may make them more inclined to adopt heuristic information-processing models, ignore key information revealing fraud and thus fall victim to online fraud (Han et al., 2016). Admittedly, even though most studies argue that cognitive ability may be a factor in online cheating toward older adults, there were dissenting voices (Phillips et al., 2008; Ross et al., 2014; DeLiema et al., 2016). DeLiema et al. (2016) found that there were few significant differences in cognitive functioning at the time victims' assets were taken, although their social contexts were different.

Personality traits are also an important area of the susceptibility of older adults to online fraud. Personality factors often influence the interactions between the victim and the fraudster (Ashton and Lee, 2008; Evans and Lee, 2014). However, the personality traits that make people more likely to respond to online fraud are currently unknown. For example, people with a pleasant personality may be more likely to trust others; therefore, they are more inclined to adopt heuristic information-processing mechanisms and are thus more vulnerable to fraud. Compared to others, older adults may be more likely to trust others (Koole et al., 2001; Whitty, 2018; Carstensen and Hershfield, 2021). However, the relationships between older adults and trust, pleasant personality and trust, and the adopted information-processing mechanism and trust are unclear (Carter and Weber, 2010; Castle et al., 2012; Han et al., 2016; Shao et al., 2019).

In addition, it must be mentioned that these research conclusions are only part of the research results, and some studies have reached the opposite conclusion (Ross et al., 2014). For example, studies have shown that increased loneliness among older adults will prompt them to more actively communicate with the outside world, respond more actively to fraudulent information, and become more likely to be targets of online fraud (Alves and Wilson, 2008). However, scams carried out on older adults using loneliness are more common in face-to-face scams than in online scams (Huang, 2020; Deng, 2021). Therefore, the impact of loneliness on the susceptibility of older adults to online fraud is uncertain.

#### Level of experience

Rich experience and knowledge can often improve the financial risk perception of fraud targets and help them identify scams (Gavett et al., 2017). With age, the accumulation of experience can compensate for the defects caused by the decline in cognitive ability (Czaja et al., 2006), and sufficient knowledge reserves can enable older adults to make more appropriate decisions using the heuristic information-processing mechanism (Li et al., 2013). Some studies have shown that the effect of empirical knowledge in preventing older adults from being deceived may be greater than that of cognitive ability (James et al., 2012; Ross et al., 2014). Will the difference in experience and knowledge affect the susceptibility of older adults to online fraud? Older adults tend to have richer experience with fraud but lack internet use experience (Czaja et al., 2006), but research has found that rich internet use experience can reduce susceptibility to online fraud (Diao and Zeng, 2020). However, whether the experiences of victims of fraud can compensate for the shortcomings of internet experience is still unknown. Other studies found no age differences in overall classification accuracy, regardless of network experience (Sarno et al., 2020).

#### Techniques of fraudsters

The widespread sending of phishing emails may seem untargeted, but it uses deliberately set signs and terms that can affect victims' susceptibility (Fischer et al., 2013). From this information, we can extract some factors that affect susceptibility to online fraud, such as stimulating human instincts through the offer of high rewards (Ariely et al., 2009; Kircanski et al., 2018); however, how these factors work is also unknown. Previous research found that the fraud techniques used by scammers are mainly focused on stimulating the victim's instincts (such as fear and greed). Instinctive information processing affects the victim's perception of clues that might otherwise trigger suspicion and affect people's decision-making. The mechanism makes victims highly motivated to reply or click on the link (Langenderfer and Shimp, 2001; Fischer et al., 2008; Kircanski et al., 2018). It is generally believed that high instinct factors produce high motivation (Wang et al., 2012), but some studies have shown that motivation and instinct factors are not synchronized and that the influence of instinct factors does not always become stronger when motivation is stronger (Frey, 1986; Ariely et al., 2009). We also do not know much about the relationship between motivation and instinct factors and the mechanism of motivation and instinct factors in susceptibility to fraud.

## Limitations

### Research method of online fraud toward older adults

The selected papers might be criticized as non-representative, and the research content might be considered irrelevant due to the choice of the keywords and the limitation of the database, which could produce doubts about our selection criteria. The current methods used to research victims of online fraud among older adults are mainly simulation experiments and questionnaires. As previously shown, laboratory simulations of deception investigations have low reliability in their conclusions because when participants believe that their behavior is being monitored, they act in line with social expectations or investigators' expectations rather than in accord with their actual thoughts. The results of the questionnaire could vary with content and focus.

In future research, researchers could refine the content of the questionnaire to make it more targeted. Future investigation of online fraud victims could combine questionnaires and interviews and track the participants longitudinally to monitor their performance more accurately. Previous research usually used lists of victims provided by complaint agencies and other official authorities from which to recruit participants. To obtain sufficient samples, older adults who were defrauded long ago were also included in the scope of the investigation (Ross et al., 2014). However, as age increases, the memory of older adults becomes poorer, and as mentioned above, their memories of negative events might be vague (Carstensen and Hershfield, 2021). Therefore, future research should consider the timeliness of the memory of fraud victims. Future researchers could investigate the memory ability of older adults to determine the time range for the most accurate memory recall among older fraud victims and select more targeted participants. Finally, different types of fraud toward older adults (such as grandparent scams, romance scams, phishing, and social networking fraud) may be affected by different psychological factors. In future studies, psychological factors can be discussed for specific types of fraud.

In addition, the phenomenon of internet fraud is also a relatively sensitive issue. Respondents tend to hide the real situation or attitude, resulting in systematic social desirability bias in the survey results of occurrence frequency and the attitude recognition degree of fraud victims (Zerbe and Paulhus, 1987). For desirability bias, the implementation of social surveys can be changed, such as by using the computer-assisted self-administration interview (CASI). Compared with face-to-face interviews, the CASI can more effectively improve the willingness of victims to admit online fraud (Gonzalez-Ocantos et al., 2012). The list experiment method can be used to indirectly ask sensitive questions by nesting a sensitive answer into the options in the questions, and the interviewees may avoid the problem of social expectation deviation that may result from direct answering (Zuo, 2020).

### Research content of online fraud toward older adults

It should be noted that our research lacks countermeasures to prevent and control online fraud of older adults. Coping strategies are also not mentioned much in the research conclusion. On the one hand, the literature included in our study (using experiments and questionnaires) is more theoretical, and the prevention and control strategies are based on their own research: DeLiema et al. (2016) proposed that reducing loneliness and increasing social networks may help protect older people from online fraud; Ebner et al. (2020) asserted that verbal fluency and positive affect in middle-old age may contribute to resilience against online spear-phishing attacks; and Gavett et al. (2017) claimed that educational background and prior phishing knowledge protects seniors from phishing. However, there is no consensus on prevention and control strategies based on their research. On the other hand, the test of the effect of countermeasures relies more on experimental methods, and the experimental methods included in our analysis involved the control of experimental conditions (such as time pressure, Sarno et al., 2020). Different from medical meta-analyses, they analyzed the results of various intervention strategies to verify the intervention effect.

Prevention and control strategies for older adult online fraud victims should be the focus of future research. However, the first prerequisite should be to further clarify the factors that make older adults susceptible to fraud. Specifically, control experiments should be carried out through intervention experiments to observe and test the intervention effects of prevention and control strategies under experimental conditions. It should be further noted that intervention strategies are a long-term process. In theory, older adults who have suffered online fraud have experience in fraud prevention and control and should be cautious in making decisions when facing online information. Previous studies have also shown that experience plays a role in fraud prevention and control (Ross et al., 2014; Sarno et al., 2020). However, it is confusing that some older adult victims repeatedly fall into the trap of fraud (James et al., 2012; Button et al., 2014). Therefore, research on the effectiveness of prevention and control strategies for online fraud toward older adults needs not only to be tested in experimental conditions but also to be verified by a tracking method.

## Implications of the systematic review

Compared with other groups of fraud victims, older adults not only suffer property losses (Kircanski et al., 2018) but also may experience new or intensified negative emotions and mental disorders, such as depression (Button et al., 2014), thus rendering them doubly vulnerable to becoming victims of fraud once again, which forms a vicious cycle (James et al., 2012). Therefore, online fraud victimization among older adults is a social and public health issue that deserves attention (Lichtenberg et al., 2016; Yan et al., 2021).

At the theoretical level, we raised a worthy question about whether older people are common victims of internet fraud or uniquely susceptible to such fraud. This is a precursor question for follow-up research. Susceptibilities may vary across groups. The susceptibility of teenagers may be related to factors such as a lack of experience and a simple living environment (Li and Qu, 2020), and older people also have specific predispositions related to being deceived. Based on a systematic review of previous research on online fraud toward older adults (such as investigating research theories, objects, methods, etc.), previous studies on the susceptibility of older adults to online fraud mainly focused on three aspects: psychology, experience and fraud techniques. The core was cognitive factors. Although current theoretical researchers have not reached a consensus on the above research, our study could provide theoretical guidance for subsequent research.

At the practical level, the current selection of psychological factors for online susceptibility and their conclusions are largely based on anecdotal evidence and biased data (Nelson, 2004; Urbina, 2012; Ross et al., 2014). This phenomenon could further cause the formulation of misguided prevention policies, which results in a waste of resources and a higher prevalence of crime. Our research held a cautious attitude toward research on prevention and control strategies for online fraud toward older adults. This is mainly because there is no unified conclusion on the factors influencing deception in older adults. However, one of the goals of our study is to remind policy-makers not to rely on the results of a single academic study but rather to adopt tried and true strategies when formulating policies to prevent and control online fraud toward older adults.

## Conclusion

There is no evidence that older adults are more vulnerable than other groups to online fraud. Current researchers are focusing more on why older adults are easy targets for fraud. The susceptibility of older adults to internet fraud may be different from that of other groups and mainly related to their cognition, trust, internet experience and the technology used by fraudsters. However, there is no consensus on the mechanism of the effect.

## Data availability statement

The original contributions presented in the study are included in the article/Supplementary material, further inquiries can be directed to the corresponding authors.

## Author contributions

YS: ideas, data collection, writing, and revisions. BM: writing and revisions. ZW: revisions. XD: analysis and revisions. MC: ideas and data collection. All authors contributed to the article and approved the submitted version.

## Funding

This work was supported by Legal Construction and Legal Theory Research Project of China (Grant no. 20SFB4038).

## Conflict of interest

The authors declare that the research was conducted in the absence of any commercial or financial relationships that could be construed as a potential conflict of interest.

## Publisher's note

All claims expressed in this article are solely those of the authors and do not necessarily represent those of their affiliated organizations, or those of the publisher, the editors and the reviewers. Any product that may be evaluated in this article, or claim that may be made by its manufacturer, is not guaranteed or endorsed by the publisher.
