# Supplementary material for: The psychology of the internet fraud victimization of older adults: A systematic review
Source: Front Psychol. 2022 Sep 5;13:912242. doi: 10.3389/fpsyg.2022.912242 (PMC9484557; doi:10.3389/fpsyg.2022.912242)
Supplement: Supplementary file 1 [file Table_1.docx]

| **A literature review of older adult victims of Internet fraud** | | | | | |
| --- | --- | --- | --- | --- | --- |
| **Authors** | **Year** | **Location** | **Method** | **Sample** | **Key findings** |
| Han et al. | 2016 | USA | Self-report and  Experimental | Old adults(n=730) | Older people with mild cognitive impairment may be more gullible than cognitively normal older adults |
| Ross et al. | 2014 | Canada | Experimental | General population, complainants, victims | The effect of cognitive decline on deception in older adults was less significant than previously thought, and risk aversion increased with age |
| Boyle et al. | 2012 | USA | Scales | Non-dementia patients from the Memory and Aging Project(n=420) | The faster the cognitive decline, the worse the decision-making ability, the more gullible |
| Reisig et al. | 2009 | USA | Telephone data analysis and Scales | Adult Internet users in Florida (n=573) | Perceptions of risk are disproportionately affected by physical vulnerabilities; Consumers with higher risk scores spent significantly less time on the Internet and made fewer purchases online, while financially impulsive respondents did not adopt this risk-reducing strategy |
| Lichtenberg et al. | 2013 | USA | Scales | Old people(n=4400) | Levels of depression, financial satisfaction and satisfaction with social needs were important predictors of deception among older adults |
| Alves & Wilson | 2008 | USA | Scales | Old people(n=28) | Decline in health and loss of intimacy can increase loneliness in older adults, and increased loneliness can make older adults more susceptible to fraud |
| Harrison et al. | 2016 | USA | Experimental | undergraduate students(n=200) | High-level of communicative doubt increases uncertainty and leads to a desire for more information before making a judgment. The desire for more information leads to systematic processing of available information and more accurate phishing spoofing detection |
| Judges | 2017 | Canada | Scales | older people' victims and non-victims(n=174) | older people' victims scored lower on measures of cognitive ability, honesty-humility and conscientiousness |
| Holtfreter et al. | 2008 | USA | Scales | A representative sample of Florida adults(n=922) | Remote buying activity increases the risk that consumers will be targeted by fraud Low self-control has no effect on whether consumers will be targeted, but it significantly increases the likelihood that they will be victimized by fraud |
| Gavett et al. | 2017 | USA | Experimental | University student(n=91);  Old people(n=102) | Educational background and prior phishing knowledge will protect seniors from phishing |
| Czaja et al. | 2006 | USA | Experimental | Individuals (n=1204) | the older adults were less likely than younger adults to use technology in general, computers, and the World Wide Web. The results also indicate that computer anxiety, fluid intelligence, and crystallized intelligence were important predictors of the use of technology |
| Stanley et al. | 2008 | USA | Experimental | Young and older adults (n = 364) | For crime interviews only, reduced emotion recognition was related to poor deceit detection for older adults in the visual condition |
| Whitty et al. | 2018 | UK | Scales | Participants(n=12060) | The psychological characteristics of romance scam victims by comparing romance scam victims with those who had never been scammed by MMFs. Romance scam victims tend to be middle-aged, well-educated women. Moreover, they tend to be more impulsive (scoring high on urgency and sensation seeking), less kind, more trustworthy, and have an addictive disposition |
| DeLiema et al. | 2016 | USA | Self-report and  Experimental | Older adult mistreatment cases(n=924) | Fraud and financial exploitation victims performed poorly on tests of cognitive functioning and financial decision making administered by a forensic neuropsychologist following the allegations. Based on retrospective record review, there were few significant differences in physical health and cognitive functioning at the time victims’ assets were taken, although their social contexts were different. Significantly more fraud victims were childless compared with financial exploitation victims. Fraud perpetrators took advantage of elders when they had no trustworthy friends or relatives to safeguard their assets |
| Kircanski et al. | 2018 | USA | Experimental | Older adults ages 65 to 85 years (n=100)  Younger adults ages 30 to 40 years (n=115) | Fraud susceptibility was assessed through participants' responses to misleading advertisements. Both HAP and HAN emotions were successfully induced in older and younger participants. For participants who exhibited the intended induced emotional arousal, both the HAP and HAN conditions, relative to the LA condition, significantly increased participants' reported intention to purchase falsely advertised items |
| Doocy et al. | 2001 | USA | Self-report and  Experimental | Salespersons (n=162) | This paper uses a variety of research tactics to portray  telemarketing fraudsters and the ruses that they employ in their work. It also seeks to locate the behavior within the typological family of criminal behavior and to suggest some possible means of reducing fraudulent telemarketing activities |
| Ebner et al. | 2020 | USA | Experimental | Young users (n=100)  Young-old users (n=41)  Middle-old users (n=16) | Short-term memory, verbal fluency, and positive affect in middle-old age may contribute to resilience against online spear-phishing attacks. The results inform mechanisms of online fraud susceptibility and real life decision supportive interventions towards fraud risk reduction in aging |
| Shao et al. | 2019 | China | Scales | Chinese older adults (n=254) | Credulity, rather than general trust, is a risk factor in vulnerability to fraud among older adults, and may inform the development of supportive interventions to reduce this population’s risk of falling victim to fraud |
| James et al. | 2014 | USA | Scales | Community-dwelling older adults without dementia (n=639) | Older age and lower levels of cognitive function, decreased psychological well-being, and lower literacy in particular may be markers of susceptibility to financial victimization in old age |
| Sarno et al. | 2020 | USA | Experimental | Younger adult(n=10)  Older adult(n=10) | Older adults appear to be more cautious when classifying emails. However, being extra careful may come at the cost of classification speed and does not seem to improve accuracy |
| Lichtenberg et al. | 2016 | USA | Experimental | Participants (n=52) | Results indicate that fraud victimization among older adults is rising, and that vulnerability variables, along with some demo- graphic variables, predict new cases of fraud. |
